# State Board of Health

**Published:** 1899-12

**Authors:** 


### STATE BOARD OF HEALTH.

IT now seems probable that Georgia is to have the long-hoped for State Board of Health. The Legislature which is now in session will probably pass the bill known as the Speer Bill, it having been introduced at the request of Governor Candler. We give below the bill in full:

SECTION 1. Be it enacted by the General Assembly of the State of Georgia, and it is hereby enacted by authority of the same, That within thirty days from the passage of this act the Governor shall appoint a State Health Commissioner by and with the consent of the Senate.

SEC. 2. Be it further enacted, That the Commissioner of Health shall be a regular reputable physician, skilled in physiology and sanitary science, and he shall be chief health officer for the State of Georgia, who shall hold his office for a term of four years.

SEC. 3. Be it further enacted, That the Commissioner of Health shall have jurisdiction over all local and county health authorities in this State, and shall formulate and promulgate under the direction of the Governor rules and regulations regarding the public health and sanitation of the State, and shall formulate rules and by-laws for the purpose of suppressing contagious or infectious diseases in this State and the abatement of nuisances and the enforcement of quarantine and isolation in cases of epidemic diseases in this State. He shall have full power in all cases where he shall, by the advice and consent of the Governor, deem it necessary to quarantine against any infectious or contagious diseases, and shall, by and with the consent of the Governor, formulate and promulgate such rules, by-laws and regulations as he may deem necessary, and provide for the enforcement of the same in this State or sections of the State, as from time to time may be deemed necessary; provided, that nothing herein shall conflict with the maritime quarantine which is left to the charge and direction of the national government.

SEC. 4. Be it further enacted, That the Commissioner of Health shall appoint by and with the approval of the Governor such subordinate officers as may be necessary to perform the duties of the office.

SEC. 5. Be it further enacted, That there shall be and is hereby created the office of county health officer in each county of this State. The said county health officer shall be a regular reputable physician who is versed in sanitary science and licensed and in active practice. The ordinaries of each county shall appoint said county health officer, who shall hold his office until the grand jury of their respective counties, which grand juries of each county, at their first meeting after the passage of this act, shall recommend some reputable licensed and practicing physician, and upon such recommendation the Governor shall appoint such county health officer, who shall hold his office for the term of four years.

SEC. 6. Be it further enacted, That upon the failure of the grand jury to recommend the appointment of the county health officer, the State Health Commissioner shall appoint some reputable physician in active practice in the county, who shall hold his office until such recommendation is made, unless removed by the State Health Commissioner.

SEC. 7. Be it further enacted, That the sum of $3,000 is hereby appropriated annually for the current expenses of the health department and payable from any unappropriated money in the treasury of the State, and the Governor is hereby authorized to draw his warrant upon the treasury for the payment of the same, and the Governor is further authorized by this act that in case of epidemic or epidemic caused from any contagious or infectious disease, to draw his warrant upon the treasury for the purpose of providing funds for the suppression of said disease.

SEC. 8. Be it further enacted, That all moneys of the department of health shall be expended by the Commissioner of Health under the direction of the Governor, and the Commissioner of Health is hereby required to render vouchers in duplicate for all such expenditures, one copy of which shall remain on file in his office, the other on file in the office of the Treasurer of the State, and for all moneys paid the Commissioner of Health shall render a receipt from the payee as a sub-voucher.

SEC. 9. Be it further enacted, That the salary of the county health officer shall be fixed by the county commissioners of each county in this State, except in counties where there are no county commissioners, in which event the ordinary shall fix said salary, and they are hereby required by the 1st day of July, each year after the passage of this act, to draw their warrant upon the county treasurer of each county in the State for the payment of the amount of such services rendered.

SEC. 10. Be it further enacted, That the State Health Commissioner shall be paid the sum of $2,000 salary per annum out of the amount herein appropriated, and the Governor is hereby authorized to draw his warrant upon the Treasurer for the payment of the same; and the Governor is further authorized to fix the salary of the clerical force of said department, not to exceed $1,000 per annum, and is hereby authorized to draw his warrant upon the Treasurer for the payment of the same.

---

THE late Phineas T. Barnum and the sempiternal Mary Baker G. Eddy deserve a niche in the temple of fame as the ones who reduced the eternal gullible to something like a system. Both were past, the one decidedly past, masters in the art of working the people; both understood how to turn the axiom, "the people want to be deceived, therefore let us deceive them," into dollars. They appealed to the imaginative side of man, and from their own standpoint they said that it was good. The one knew that the man is but the child grown up; the other, that man is essentially idolatrous and fetich-worshiping and that the mass of humanity had rather be fooled than shown its folly. It was Barnum's hobby to have a "What is it," an acme of mystification, an incarnation of a creature that hitherto existed only in the imagination.

Eddyism is much the same sort of a "What is it?" and invites the exact belief that would give credence to a mermaid and a missing link.

The caged "What is it?" of the showman was found to be a blind, microcephalic idiot, and Mrs. Eddy's peculiar "science" is becoming so much of a joke that it will ere long be laughed out of court like the idiot was out of the side-show.

The only serious side of Christian science is that it sometimes kills its devotees, or more often and more pitifully the children of the peculiar people. But for this phase of it, which is being legally dealt with, the cult is no more worthy of consideration by sensible people than any other worn-out hoax.

Faith-cure, Weltmerism, Dowieism, magnetic healing and the like differ from Christian science in that they possess a grain of truth and are founded upon the well-known, if somewhat overworked, therapeutic resource, suggestion.

When it is remembered how great is the influence of mind over the body and its ailments, and how many people suffer from *la maladie imaginaire,* we can readily understand the rationale of the cures effected by these so-called systems. It is suggested that the patient be well and at once his mind attaches itself to the new and cheerful thought substituted for his old hopelessness, and he is raised out of his slough of despond and restored triumphantly to health, "after all the doctors had failed." Furthermore acute diseases tend to get well, and the cure is not due to medicine in any application of the word or the thing, but to entirely natural causes. Faith-cure and its congeners step in here and vociferously claim the cure. Is faith the master of nature, or nature of faith? Was ever a gross pathological lesion brought to the normal by prayers and genuflexions, and is magnetism powerful enough to draw out a bit of steel buried in the eye? "Canst thou draw out the leviathan with a hook? Canst thou speak soft words unto him?"

The people want to be deceived, that is the *crux* of the whole matter. They lend an attentive ear to any one who has anything marvelous to tell about their bodies and the diseases that affect them. An average layman's knowledge of anatomy and physiology is about equal to that of the Chinese. He may hesitate to believe that the moon is made of green cheese, but he will carry a buckeye in his pocket to ward off the rheumatism and have his child wear amber beads to prevent the croup, and will give sober credence to the wildest fictions of charlatanry. No bank is so small but has its depositors, no theory is so fanciful, absurd and puerile but has its adherents.

It has been so since there were people enough on earth to strike a balance of human nature, and it will continue so long as men work, laugh, love and suffer.

Magnetic healing and the like (the name is of no consequence) is but a strand in the varicolored fabric of society and civilization. Draw it out and it leaves a dead space that is sure to be filled in by something that fits it.

If it were viewed from the proper altitude, its real meanness of proportion could be seen, but we look at it too close and on a level. Then only does it bulk as a competitor.

If there be truth in any of these beliefs it will prevail, and all the husk that envelopes it be swept into the rubbish heap.

Mesmer was the forerunner of hypnotism, Hahnemann of small dosage. In both instances that which was true has come to the surface and survived—the untrue has been drowned in oblivion. The same fate will inevitably overtake all untried, unreasonable beliefs, with the exception of Christian science, which awaits total annihilation, and has existed so far merely from a blasphemously alleged connection with the religion of Christ and the Book wherein it is revealed.

---

A CONTRIBUTOR to the *Medical Brief* pays this compliment to Homeopathy: "Hahnemann, with his Homeopathy, and later Scudder with his specific medication, have taught their followers to go direct from symptoms to remedies. Every symptom indicates a drug and every group of symptoms has somewhere its remedy. The truth to which the old school fogies are now awakening is that it is possible to cure a disease without stopping to name it, and that by studying the relations between symptoms and remedies, diseases can be cured without resorting to 'shot-gun' methods."—*The Critique.*

This is tantamount to saying that the disease should be disregarded, symptoms alone are to be considered. Now, what is a symptom? It is any affection which accompanies disease; a perceptible change in the body or its functions which indicates the disease or the kind or phases of disease. A symptom is, therefore, essentially dependent upon something else, and has no independent existence. If it exists alone, it ceases to be a symptom and becomes the disease itself. It is necessary for study and description and identification that a disease should have a name, and symptom-complexes receive special designations. Otherwise confusion would reign and the result would be something like the Chinese alphabet—interminable. It is our constant aim, by both methods of reasoning to arrive at conclusions. These conclusions, if the last reduction be correct, we take to be an integer and so name it. This simple reasoning is plain enough to all except those who are so steeped in ignorance and sunk in the mud of bigoted sectarianism that their eyes are covered and their ears are plugged. Then again there are fools, who are to be judged according to their folly.

Suppose (and this is addressed to the ignorant, not the fool) all treatment were symptomatic, and there was nothing said of disease, pathological lesion or cause of it, where would it lead us? Pain in a joint, would be simply pain in a joint, whether due to rheumatism, tuberculosis, osteomyelitis, arthritis, synovitis or injury. Pain is the symptom, and the indication is for treatment of pain, nothing further. A chill is a symptom, and needs treatment, it matters not whether it be due to the onset of an acute inflammation, an infectious disease, hemorrhage, malaria or the influence of cold. Difficult breathing is a symptom. Give the remedy for difficult breathing, and the indications are fulfilled, whether it be due to laryngismus stridulus, membranous croup, pneumonia, edema of the lungs, cardiac disease, or tight corsets, or a full belly. And so on, multiplying examples until the mind reels before the vastness of the "symptomatology" and the possibilities for doing harm vested in blind adherence to a mad creed "that goes direct from symptoms to remedies." "It is possible to cure a disease without stopping to name it," naturally, but it is none the less a disease, and the lack of a name for it is an acknowledgment of ignorance. It is just such ignorant, disloyal men as the contributor to the *Medical Brief* who stand in the way of progress and bring discredit upon the medical profession.

